# Effects of Chlorantraniliprole on Oxidative Stress, Enzymatic Biomarkers, and Hepatic Transcriptome in *Alosa sapidissima* (Wilson, 1981)

**DOI:** 10.3390/ijms27125383

**Published:** 2026-06-15

**Authors:** Yao Zheng, Noa Shapumba, Gangchun Xu

**Affiliations:** 1 Wuxi Fisheries College, Nanjing Agricultural University, Wuxi 214081, China; noa.shapumba@gmail.com; 2 Key Laboratory of Freshwater Fisheries and Germplasm Resources Utilization, Ministry of Agriculture and Rural Affairs, Freshwater Fisheries Research Center (FFRC), Chinese Academy of Fishery Sciences (CAFS), Wuxi 214081, China

**Keywords:** American shad, chlorantraniliprole, histological slice, oxidase, transcriptome

## Abstract

The purpose of this study was to investigate the adverse effects of 1.5 μg·L^−1^ environmentally relevant chlorantraniliprole (CAP) on oxidase biomarkers (juvenile, 2.5 g) for 2, 4, and 8 h and transcriptomic response (adult, 254.8 g) for 96 and 192 h in American shad *Alosa sapidissima* (Wilson, 1981). American shad is sensitive to pollutants and has become an important economic fish in China, especially for recirculating the aquaculture system and photovoltaic farming. For juvenile shad under short-time CAP exposure, acid phosphatase (ACP) and aryl hydrocarbon receptase (AHR) at the protein level significantly increased at 2 h, and for longer-time exposure, alkaline phosphatase (AKP), polyphenol oxidase enzyme (PPO), and tumor necrosis factor alpha (TNFα) at the protein level significantly decreased; ryanodine receptase (RYR) at the protein level was significantly increased at 8 h. Interestingly, malondialdehyde (MDA) contents, biomarkers of oxidative stress, were significantly decreased for depletion at 2 h and 4 h, while they increased for eliminating free radicals at 8 h via longer-time CAP exposure duration. With the same CAP exposure for adult shad, the number of congested and dilated sinuses of the liver changed, with fine granular brown pigmentation and vacuolization of hepatocytes at 96 h, while the sinuses and central veins were dilated and edematous degeneration occurred at 192 h for longer-time exposure. The detected enzymatic activities, except for adenosine 5′-monophosphate (AMP)-activated protein kinase (AMPK), significantly decreased, and MDA contents significantly increased in adult shad at 96 and 192 h. Ribosome, proteasome, spliceosome, protein processing in endoplasmic reticulum, oxidative phosphorylation, glycerophospholipid metabolism, biosynthesis of amino acids, ferroptosis, peroxisome, apoptosis, necroptosis, and mTOR signaling pathways were the most significantly enriched pathways. For qPCR verification, the genes *ppa2*, *pla1a*, *psmb13a*, *pkz* and *stat1b* were significantly upregulated, while *hspa8b*, *capn2*, *tram2*, *asns*, *bcl2l1*, *diablo*, and *prkcb* were downregulated in adult shad. The results reveal elevated oxidative stress causing time-dependent hepatic damage via 1.5 μg·L^−1^ CAP exposure both in juvenile and adult shad.

## 1. Introduction

Bisamide insecticides occupy a huge share in the pesticide market and are commonly used in the integrated rice–fish farming system. Their widespread agricultural use has led to increased research attention toward their toxicity, potential ecological risks, and residue accumulation in aquatic environments. Chlorantraniliprole (CAP) is a synthetic insecticide that belongs to the anthranilic diamide class of compounds and is used for the control of various insect pests in crops, ornamental plants, and lawns. Contamination of water by pesticides is mainly brought about through agriculture, along with surface runoff and subsurface drainage. Its discharge into aquatic ecosystems is leading to a significant level of aquatic contamination, with potential detrimental effects on the aquatic environment and aquatic life [1,2,3]. The effects of CAP on aquatic life have been demonstrated in studies on *Channa punctatus* (96 h LC_50_ = 14.424 mg·L^−1^) [2], zebrafish *Danio rerio* (LC_50_ > 100 mg·L^−1^) [4], tilapia [5], catfish *Clarias gariepinus* [6], *Cirrhinus mrigala* [7], crucian carp *Carassius carassius* (96 h LC_50_ = 74.824 mg·L^−1^) [8], and *Labeo rohita* [2]. The 96 h LC_50_ for CAP on fish species was reported as 12 mg·L^−1^ in a review of a rice–prawn concurrent system [9]. All these existing fish and shrimp LC_50_ results indicate that the potential environmental risks of CAP to aquatic organisms should receive more attention [4], and CAP exposure could significantly disrupt behavior, leading to immunotoxicological [6] and metabolic [4] disorders.

Such reports showed that CAP, with an assumed half-life of 1000 days in water at pH = 7 and 20 °C [9], detected in the aquatic environment, can harm the haemato-biochemical profile, growth, physiology, reproduction, and immunity and cause significant histopathological changes in fish [2,4,5,6,7,10,11]. Consequently, certain antioxidant, neurotoxic, macromolecular and metabolic enzymes are identified to be affected by specific pollutants in fish, forming oxidative stress enzyme biomarkers [12], and are being used to detect the presence of pollutants in water as a supplement or a substitute for general water chemistry. Enzymes, such as alkaline phosphatase (AKP) and acid phosphatase (ACP), polyphenol oxidase enzyme (PPO), ethoxyresorufin-o-deethylase (EROD), tumor necrosis factor α (TNF-α, a kind of inflammatory cytokine), malondialdehyde (MDA, a biomarker for lipid peroxidation oxygen free radicals), have been reported [4], and aryl hydrocarbon receptase (AHR) and ryanodine receptase (RYR) [13] have also been studied in our previous reports [14,15].

Recently, adenosine 5′-monophosphate (AMP)-activated protein kinase (AMPK) has also been used as a biomarker for 0.15 and 1.5 mg·L^−1^ acute CAP exposure in *Cnesterodon decemmaculatus* [12]. In addition to oxidative enzymatic assay studies, the transcriptome has also been used in understanding the effects of pesticides on fish. The hepatic transcriptome, which refers to the complete set of expressed genes (transcripts) in the liver, provides valuable insights into the liver’s function and its response to various environmental and physiological conditions. RNA-Seq is a recently developed approach for transcriptome profiling used in our toxicological studies in American shad [14,15], and it can also be used to study how fish livers respond to various environmental stressors, such as temperature changes, pollution, or nutritional imbalances, like deltamethrin exposure [16]. Hepatic mRNA-sequencing revealed only 37 differentially expressed genes (DEGs, *p*-value 0.05 and fold change ±1.5) enriched in the pathways of circadian rhythms and Ca^2+^ signaling in yellow perch (0.92 ± 0.1 g) after 0.2 µg·L^−1^ CAP exposure alone for 28 d, while 251 DEGs were reported for clothianidin co-exposure [17]. In another study of larval fathead minnows (24 h post-hatch), 7~21 DEGs (*p*-value 0.05 and fold change ±1) were found, and calcium-mediated processes, cellular communication, immune system, and nervous system pathways were significantly enriched following 0.01~0.25 mg·L^−1^ CAP exposure [18]. But, in a study of one-month-old Baikal Whitefish larvae, 242 DEGs were found with a scanning threshold of *p*-value 0.01 and fold change ±2 [19]. For post-larvae white shrimp (*p*-value 0.05 and fold change ±2), 737 DEGs were found [20]. The differences in DEG values displayed by RNA–Seq in different species make the degree of toxicity of CAP to fish unclear to researchers.

The number of DEGs was different for the same fish and shrimp species with different sizes, like white shrimp for juveniles (1.45 g) and adults (14.50 g) under virus infection, where 637 and 3700 DEGs were identified for juveniles and adults in each comparison group [21]. In a study of two *Daphnia* species (*D. mitsukuri* and *D. sinensis*) with two stages (days 2 and 8 for juvenile and adult) of exposure to the same predation threat [22], the total DEGs for juveniles and adults were 1229 and 1475 in *D. mitsukuri* but 1229 and 341 DEGs for juveniles in *D. mitsukuri* and *D. sinensis*. Such differences have also been found in fish species, like zebrafish [23] and largemouth bass [24]. American shad has a similar taste to the extinct *Tenualosa reevesii* in China. Currently, there are few reports on the negative impact of environmental pollutants on different stages of shad.

The detected maximum concentration of CAP was 34.433 mg·L^−1^ in rice ecosystems in Sri Lanka [4], and 1.35 µg·L^−1^ and 2.55 µg·kg^−1^ CAP can be found in the water and sediment samples in the rice–crab system of China, with 82.22% of the total spraying concentrations, which can also be found in the USA (10.2 µg·L^−1^) [12], Brazil [25], and Uruguay. South America was the primary market for CAP production [12]. The broader impact of CAP let researchers use high-throughput [18] and cell models to study its toxicity to different species. In the early stage, we used fish, crab and shrimp, closed shell, zooplankton and phytoplankton to investigate the 96 hLC_50_ and its toxic mechanism of CAP [26]. American shad, *Alosa sapidissima* (Wilson, 1981), is an anadromous clupeid fish native to North America and is now an important study organism, because its taste is similar to the extinct *Tenualosa reevesii* as its substitute in China because it is an emerging important ecological and aquacultural species in China with higher income (larger than 25 $·kg^−1^), especially in recirculating aquaculture systems and photovoltaic farming. Pollution may be one of the main reasons for *T. reevesii*’s extinction [27]. For ecological relevance, American shad has been found to be very sensitive to contaminants, like copper sulfate [14,15], in entering the aquatic environment [26,28], and the survival rate for the seedling stage is a big challenge, which has dropped from 90% to below 50%. In adult shad with a higher growth rate, chronic toxicity with long-time exposure may affect fish health, which may result in economic losses due to environmental pollution (like heavy metal pollution and massive Bisamide insecticide usage around the pond for shad) and bacterial infections, like *Aeromonas hydrophila* [14,28]. The objective of this study is to investigate the short- and long–time acute CAP toxicological effects, enzymatic activity in juveniles and adults, and transcriptome data to gain insights into the molecular mechanisms in adult *A. sapidissima*. The hypothesis was that CAP significantly disrupts oxidative stress and inflammation in shad in a differentiated, dose-dependent manner in juvenile and adult shad.

## 2. Results

### 2.1. Enzymic Biomarkers in the Whole Body of Juvenile American Shad

**Figure 1:**
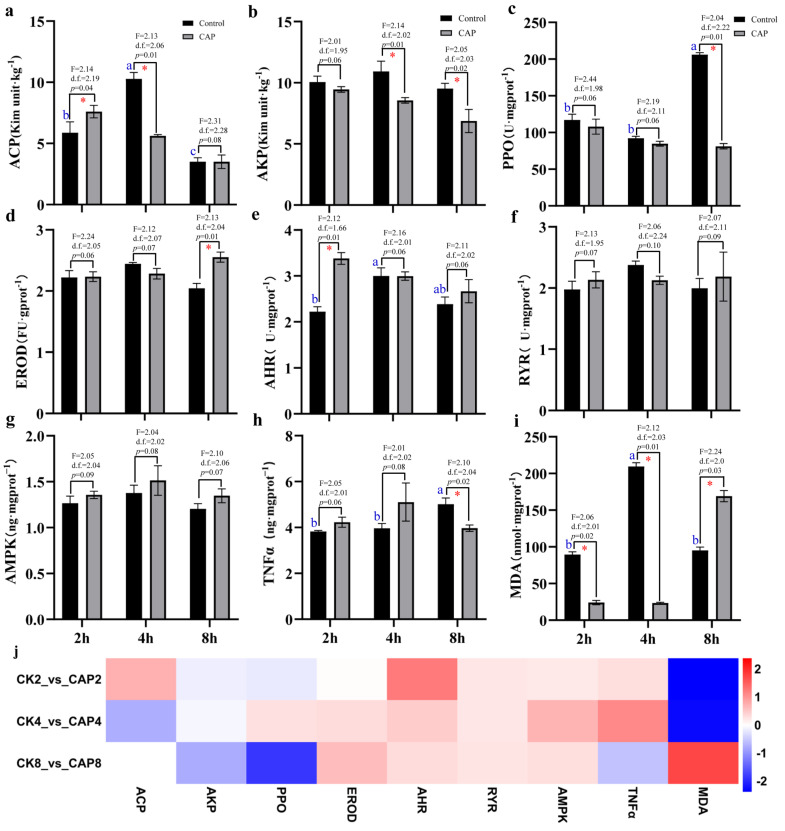
Enzymatic biomarkers in the whole body of American shad exposed to 1.5 μg·L^−1^ CAP (*n* = 3). (**a**) acid phosphatase, ACP, (**b**) alkaline phosphatase, AKP, (**c**) polyphenol oxidase enzyme, PPO, (**d**) 7-ethoxy-3-isophenoxazolone-deethylase, EROD, (**e**) Aryl hydrocarbon receptase, AHR, (**f**) ryanodine receptase, RYR, (**g**) adenosine 5′-monophosphate (AMP)-activated protein kinase, AMPK, (**h**) tumor necrosis factor alpha, TNFα, (**i**) trace malondialdehyde, MDA. The asterisks on the plot represent the significance level. The annotations for groupings are the same for each enzyme activity and shown in the last one, while 2, 4, and 8 h are only shown in the last row. (**j**) Fold changes for the detected enzymes when compared to the controls for data after normalizing, CK and CAP stand for treatments, and 2, 4, 8 stand for sampling time points. One-way ANOVA is used to compare differences at different times in controls and is shown with different lowercase letters.

**Figure 2:**
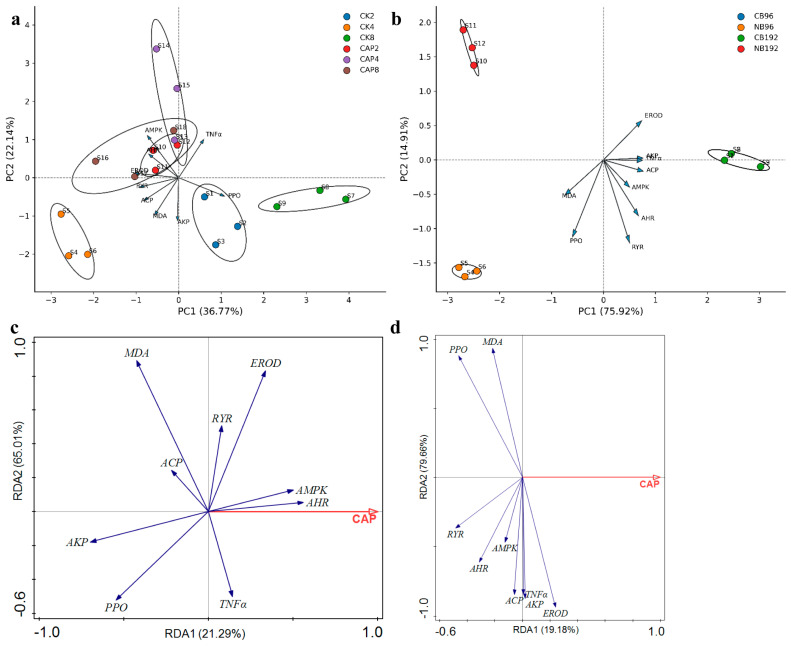
The PCA (**a**,**b**) for juvenile and adult and RDA (**c**,**d**) for enzymes by CAP exposure on the juvenile whole body (**a**,**c**) and adult liver (**b**,**d**) of American shad. a, CK and CAP stand for the controls and CAP treatments, 2, 4 and 8 stand for 2 h, 4 h and 8 h. CB and NB stand for the controls and CAP treatments, 96 and 192 stand for 96 h and 192 h.

ACP exists in the lysosomes of macrophages, catalyzes the hydrolysis reaction of phosphate monoesters, and participates in metabolic regulation and immune prevention. There was a significant increase in ACP content at the protein level after 2 and 4 h exposure to CAP when compared to the respective control groups (*p* < 0.05, Figure 1a). However, the AKP content at the protein level, a kind of phosphate monoester hydrolase, was significantly lower in treatment groups compared to control groups at 4 and 8 h (*p* < 0.05, Figure 1b). There was a low significant protein level of PPO (at 8 h), a specific marker enzymes in megakaryocytes with the function of catalyzing the oxidation of phenolic substances, in CAP-exposed groups compared to their respective control (Figure 1c). EROD activity at the protein level, relative to adaptive response, was significantly higher than the control only at the eight-hour mark of exposure (*p* < 0.05, Figure 1d). As time progressed, the AHR content at the protein level increased at 2 h, producing much more reactive oxygen species to protect against inflammation (Figure 1e). The level of RYR content at the protein level, relative to calcium homeostasis, was found to have no significant change after CAP exposure (*p* > 0.05, Figure 1f). AMPK, a key molecule regulating biological energy metabolism, showed no significant change after CAP exposure (Figure 1g), while TNFα, a pro-inflammatory cytokine secreted by macrophages and monocytes, only significantly decreased after CAP exposure at 8 h (Figure 1h). MDA at the protein level, a biomarker of oxidative stress for oxygen free radicals’ removal, was significantly lower due to exhaustion after usage of clearing oxygen free radicals at 2 and 4 h when compared with the controls (Figure 1i). Contrarily, the MDA at the protein level at eight exposure hours was significantly higher in the CAP treatment than in the controls (*p* < 0.05), indicating an increase in the protein level relative to exposure time, through the accumulation of lipid peroxidation products accompanied by sustained or aggravated oxidative stress. PCA analysis showed the enzymes in treatment groups were well separated, meaning the enzyme can be easily affected by CAP exposure (Figure 2a,b, explained 58.9%, 90.8% of the total variance). RDA analysis showed a positive relationship between CAP concentration and EROD, AMPK, AHR, RYR, TNFα but a negative relationship between ACP, AKP, PPO and MDA contents (Figure 2c). The analyses for the same treatments among different sampling time points have not been compared in this study due to the diverse expression levels at different time points. The significant increases in ACP, AHR, MDA contents (real value without standardization) at 4 h and PPO and TNFα at 8 h when compared with 2 h show the level of enzyme activity is easily influenced by the external environment and stress.

### 2.2. Hepatic Histology, Enzymatic Activities and MDA Contents in Adult American Shad

**Figure 3:**
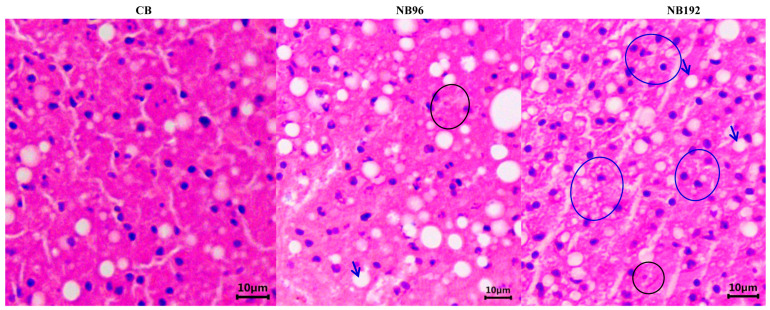
Liver tissue from American shad (H&E stain, scale bar 10 μm) (*n* = 3). Blue arrows show vacuolation; black circles indicate congested, dilated blood sinusoids; blue circles represent alteration of liver structure. CB, NB96, and NB192 stand for control, 1.5 μg·L^−1^ CAP for 96 and 192 h, respectively.

For CAP exposure at 96 and 192 h, the boundaries of hepatocytes were blurred, with indistinct intracellular structures, fine-granulated brown pigments deposition, and congested, compressed dilated blood sinusoids (black circle) shown in the present study. A significantly increased number of vacuolations of major hepatocytes (blue arrows) were found in this study (Figure 3, 8 ± 1%, 25 ± 2% and 58 ± 5% in controls, CAP exposure groups at 96 and 192 h, *p* < 0.05). Fish exposed to 1.5 μg·L^−1^ CAP for 192 h exhibited dilated blood sinusoids and central veins and significant hydropic degeneration (blue circles, 50 ± 6% of the ratio when compared to 12 ± 3% in controls, *p* < 0.05).

**Figure 4:**
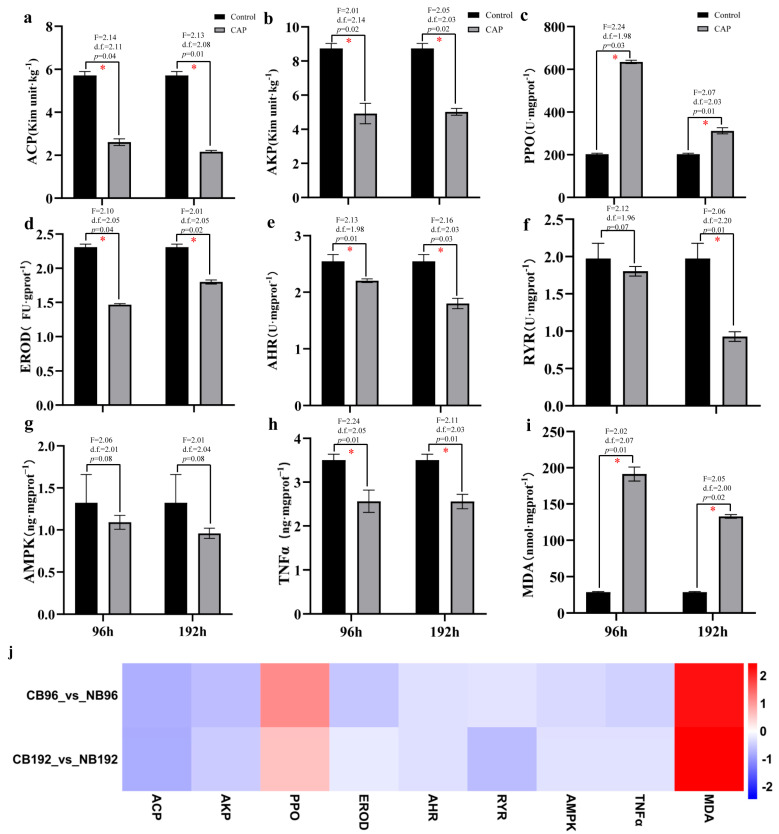
Enzymatic biomarkers in liver of adult American shad exposed to 1.5 μg·L^−1^ CAP (*n* = 3). (**a**) ACP, (**b**) AKP, (**c**) PPO, (**d**) EROD, (**e**) AHR, (**f**) RYR, (**g**) AMPK, (**h**) TNFα, (**i**) MDA. The asterisks on the plot represent the significance level. (**j**) Fold changes for the detected enzymes when compared to the controls for data after normalizing, CK and CAP stand for treatments, and 2, 4, 8 stand for sampling time points.

Enzymatic activities of ACP, AKP, EROD, AHR, RXR (only at 196 h, Figure 4), TNFα significantly decreased at 96 and 192 h, while MDA contents significantly increased, together with no significant change in AMPK. PCA analysis shows the enzymes in treatment groups were well separated without the controls, meaning the enzyme can be easily affected by CAP exposure but maintained in the controls (Figure 2b). RDA analysis showed a positive relationship between CAP concentration and EROD, AKP, TNFα but a negative relationship between other enzymes and MDA contents (Figure 2d).

### 2.3. Transcriptome and qPCR Verification in Adult American Shad

**Figure 5:**
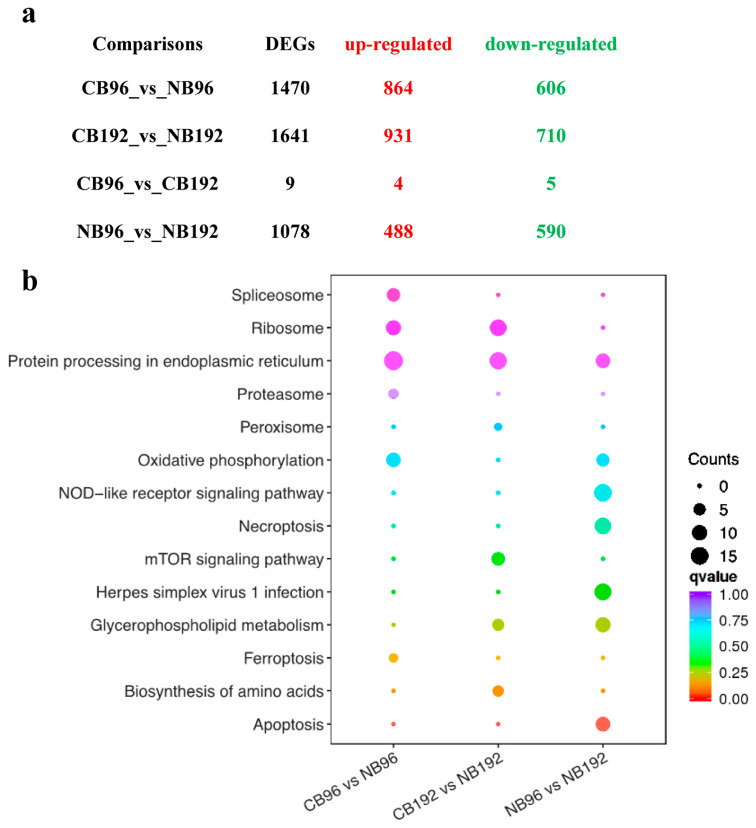
KEGG enrichment analysis of DEGs in American shad after exposure to CAP (*n* = 3). (**a**) Upregulated (red) and downregulated (green) DEGs at 96 and 192 h. (**b**) KEGG pathways enrichment of DEGs in each comparison group exposed to CAP. The comparisons were CB96 vs. NB96, CB192 vs. NB192, NB96 vs. NB192. The colors of dots on the graph represent different pathways, while the dot size represents the counts of DEGs in that pathway. CB, NB96, and NB192 stand for control, 1.5 μg·L^−1^ CAP for 96 and 192 h, respectively.

There were 1641 DEGs in total in the comparison groups of CB192_vs_NB192, and a total of 192, 150, and 127 pathways were affected in the comparison groups of CB96 vs. NB96, CB192 vs. NB192, NB96 vs. NB192, respectively (Figure 5, details in Supplementary Materials). Protein processing in the endoplasmic reticulum (ko04141) was enriched/affected in all groups, while the ribosome (ko03010) was enriched/affected in the comparison groups, both in CB96 vs. NB96 and CB192 vs. NB192. Pathways that were unique to their groups representing the difference between 96 h and 192 h exposure and their controls included proteasome (ko03050), oxidative phosphorylation (ko00190), ferroptosis (ko04216), and spliceosome (ko03040) in the comparison group of CB96 vs. NB96. Biosynthesis of amino acids (ko01230), glycerophospholipid metabolism ko00564), mTOR signaling pathway (ko04150), and peroxisome (ko04146) were shown in the comparison group of CB192 vs. NB192.

For the comparison of CB96 vs. CB192, four and five DEGs were found, and only the *lamb3* gene (laminin subunit beta-3) was significantly enriched in the ECM-receptor interaction pathway. For the comparison of NB96 vs. NB192, the NOD-like receptor signaling pathway (ko04621), apoptosis (ko04210,) and especially necroptosis (ko04217) were significantly enriched between 96 and 192 h.

**Figure 6:**
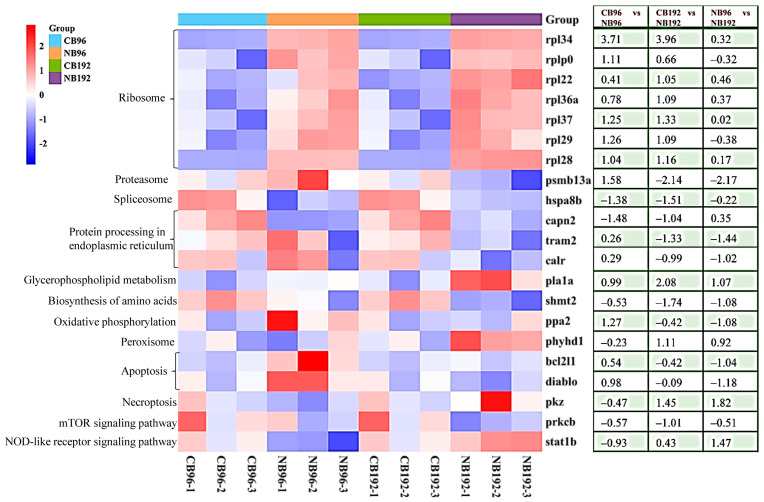
Selected DEGs in the most enriched pathways after CAP exposure (*n =* 3). CB, NB96, NB192 stand for control, 1.5 μg·L^−1^ CAP for 96 and 192 h, respectively. *rpl34*, ribosomal protein L34, *rplp0*, ribosomal protein lateral stalk subunit P0, *rpl22*, ribosomal protein L22, *rpl36a*, ribosomal protein L36a, *rpl37*, ribosomal protein L37, *rps29*, ribosomal protein S29, *rps28*, ribosomal protein S28, *psmb13a*, proteasome 20S subunit beta 13a, *hspa8b*, heat shock protein family A (Hsp70) member 8, *capn2*, calpain 2, *tram2*, translocation associated membrane protein 2, *calr*, calreticulin, *pla1a*, phospholipase A1 member A, *shmt2*, serine hydroxymethyltransferase, *ppa2*, inorganic pyrophosphatase 2, *phyhd1*, phytanoyl-CoA dioxygenase domain containing 1, *bcl2l1*, BCL2 like 1, *diablo*, diablo IAP-binding mitochondrial protein, *pkz*, protein kinase containing Z-DNA binding domains, *prkcb*, protein kinase C beta, *stat1b*, signal transducer and activator of transcription 1.

**Figure 7:**
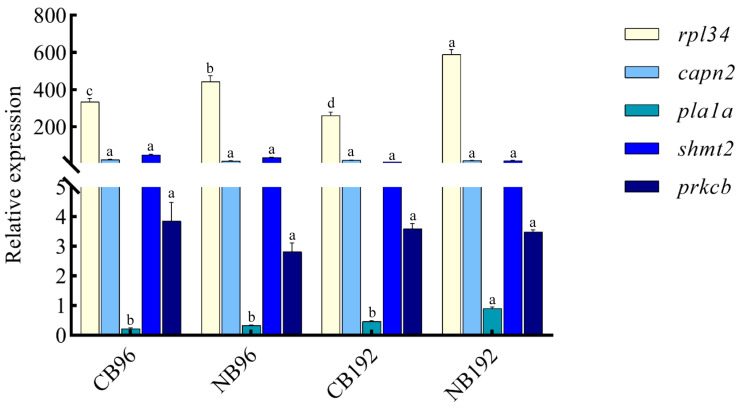
Gene verification by qPCR (*n = 3*, *p* < 0.05 stands for the significance level with different lowercase letters). *rpl34*, ribosomal protein L34, *capn2*, calpain 2, *pla1a*, phospholipase A1 member A, *shmt2*, serine hydroxymethyltransferase, *prkcb*, protein kinase C beta.

In the pathway of protein processing in the endoplasmic reticulum, *tram2* was significantly downregulated (Figure 6). The genes *capn2* (Figure 7) and *calr* were significantly downregulated. CAP caused upregulation of the ribosome pathway in both groups, CB96 vs. NB96 and CB192 vs. NB192. In the CB96 vs. NB96 group, the genes *rpl34* (Figure 6), *rps29*, *rps28*, *rplp0*, and *rpl37* were involved, and the genes *rpl35a*, *rpl22*, *rps28*, *rpl36a*, *rps29*, *rps28*, and *rpl37* were involved in CB192 vs. NB192. The shared genes *rpl37*, *rps29*, and *rps28* were common in both groups.

The gene *ppa2* (oxidative phosphorylation) was upregulated in the CB96 vs. NB96 group and downregulated in the NB96 vs. NB192 group. The gene *pla1a* (glycerophospholipid metabolism, Figure 7) was significantly upregulated in both groups. In the CB96 vs. NB96 group, the genes *psmb13a*, *hmox1a*, and *hspa8b* were upregulated in the proteasome, ferroptosis, and spliceosome pathways, respectively. In the CB192 vs. NB192 group, *prkcb* (Figure 7) and *shmt2* (Figure 7) were downregulated, and *phyh1l* was upregulated in the mTOR signaling, biosynthesis of amino acids, and peroxisome pathways, respectively.

**Figure 8:**
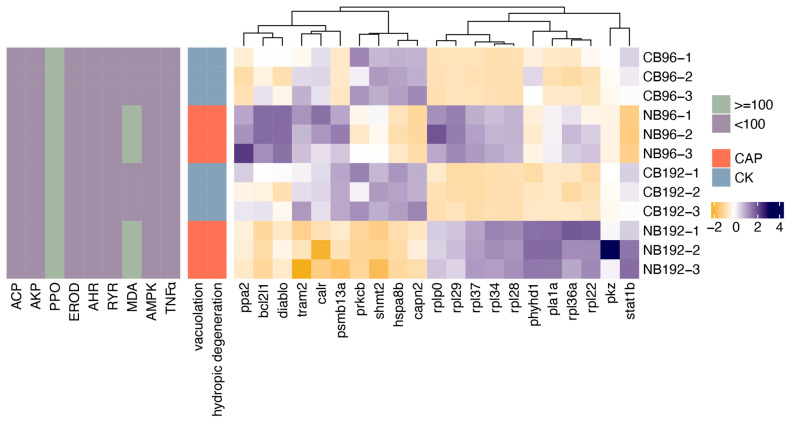
The relationship between histological changes, enzymatic activities and gene expressions after CAP exposure at 96 and 192 h. Orange and gray display CAP treatments and control groups; values > 100 show blue–green. Yellow and purple represent FPKM values of gene expression.

In the NB96 vs. NB192 group, *pkz* was upregulated in the necroptosis pathway, *bcl2l1* upregulated in the NOD-like pathway, and *diabloa* and *bcl2l1* were both downregulated in the apoptosis pathway. The qPCR verification [29] showed the accuracy with the results for the transcriptome, which may help us to ascertain the harmful effects of CAP in American shad. The histological changes had a positive effect with MDA contents (Figure 8), *prkcb*, *shmt2*, *hspa8b*, *capn2* significantly decreased, while *rplp0*, *rpl29*, *rpl37*, *rpl34*, *rpl28*, *pla1a*, *rpl36a*, *rpl22* significantly increased.

## 3. Discussion

### 3.1. Enzymic Activities and MDA Contents in Juvenile Fish

There were decreased ACP and AKP activities at the protein level found in tilapia livers in response to microcystins in a time-dependent manner [30] and decreased hepatic AKP and increased ACP activity at the protein level in *O. niloticus* exposed to sublethal concentrations of chlorpyrifos [31], which may indicate that CAP impaired the fish’s cellular ability to neutralize and eliminate the toxic compound, making the organism more susceptible to the harmful effects of the pesticide. The increased ACP may suggest that CAP disrupted the fish’s metabolism, potentially leading to reduced energy availability and other physiological impairments, which showed a negative relationship. The increase in EROD content (positive with CAP exposure) or activity at the protein level suggests that the CAP pesticide is inducing the upregulation of this enzyme, which is part of the fish’s adaptive response to metabolize and eliminate the pesticide, which has been performed in tilapia after diuron exposure [32]. In this study, the increase in AHR content at the protein level suggests that the CAP pesticide is being recognized by the fish’s AHR system [13,14,15], which was similar to the results in marine fish in Kesennuma Bay following heavy oil-derived polycyclic aromatic hydrocarbon exposure [33]. Activation of the AHR pathway can lead to the generation of reactive oxygen species and the induction of inflammatory responses. This study showed an increase without a significant level in RYR (CAP’s agonist) at the protein level [13], different from the study performed in *Pimephales promelas*. RYR is reported as being associated with neuromuscular development [13], and its increment resulting from mitochondrial dysregulation [34], which has been performed in other fish species, corresponds to the affected pathways. In this study, this suggested the disruption of calcium homeostasis occurred in American shad.

The dysregulation of calcium homeostasis can lead to the generation of reactive oxygen species and the induction of oxidative stress. Similarly, the contents of MDA at the protein level were significantly increased in *C. gariepinus* and zebrafish after exposure to CAP [4]. However, the increase in MDA content (positive with histological changes) at the protein level at 8 h suggests that the CAP exposure induced significant oxidative stress and cellular damage in the fish, which showed a negative relationship. This study’s results revealed that the MDA [4] at the protein level was higher for lengthy exposure than short exposure, like in *Astyanax altiparanae* exposed to diesel [35]. This indicates the fish’s antioxidant defense systems are being overwhelmed or impaired by CAP pesticide exposure or a reflection of the fish’s struggle to maintain energy homeostasis under the stress of CAP exposure.

### 3.2. Transcriptome and Histological Alterations in Adult Fish

Enzymatic activities in juvenile and adult shad showed different metabolic responses after CAP exposure, like PPO (decreased for juvenile and increased for adult shad), EROD and AHR (increased for juvenile and decreased for adult shad). It hinted adaptive response, oxidation resistance, and even inflammation, reflecting a different status after CAP exposure, which has been confirmed by MDA contents (which also showed a negative relationship). Interestingly, MDA contents, a biomarker of oxidative stress, were significantly decreased for depletion at 2 h and 4 h, while they increased for eliminating free radicals at 8 h via longer-time CAP exposure duration. Firstly, MDA depletion for removing free radicals in a short-duration exposure appeared; then, the enhanced lipid peroxidation reaction occurred under the status of oxidative stress or cellular damage for a longer exposure duration. Dilated blood sinusoids, central veins, and hydropic degeneration found in this study after CAP exposure, and similar histological results, were obtained in catfish livers exposed to the insecticide Voliam flexi^®^ [6]. The ribosome pathway was the most significantly affected by CAP in this study and was most present at both 96 and 196 exposure hours. Ribosomes, as cellular organelles, are responsible for protein synthesis in the process of translation. Large ribosomes (*L10*, *L37*, *L34*) and small ribosomes (*S28*, *S29*) were highly upregulated at 96 h, while large ribosomes (*L22*, *L35a*, *L36a*) and small ribosomes (*S28*, *S29*) were highly upregulated at 192 h. The results of this study indicate that among these extra-ribosomal functions, proteins linked to cell cycle (*L37*, *L34*), apoptosis (*S29*), cell proliferation (*L36a*, *L34*), and neoplastic transformation could be induced by CAP exposure since these proteins were upregulated in CAP-exposed fish.

Protein processing in the endoplasmic reticulum is a complex and highly regulated process essential for protein quality control, proper folding, and post-translational modifications, with implications for cellular function. Calpain 2, also known as *capn2*, a member of the calpain family of calcium-dependent cysteine proteases, was downregulated. *tram2* at 192 h exposure was downregulated. *tram2* is involved in endoplasmic reticulum-associated protein degradation, a process that targets misfolded proteins for degradation. *tram2* plays a role in the recognition and retro translocation of misfolded proteins from the endoplasmic reticulum to the cytosol, where they are targeted for degradation, and cells lacking *tram2* experienced a heightened unfolded protein response upon acute endoplasmic reticulum stress [36].

The necroptosis pathway is another form of programmed cell death that differs from apoptosis, a type of “clean” cell death often linked with inflammation. *pkz* (homologous to PKR) is a serine/threonine–protein kinase, an enzyme that specifically phosphorylates serine and/or threonine amino acids on target proteins. The upregulation of these kinases can indicate increased activation of the necroptosis signaling pathway, leading to programmed necrotic cell death. Additionally, this may signal a disruption in the balance between cell death and survival, potentially causing organ or systemic dysfunction in fish. A similar effect of *pkz* was observed in grass carp (*Ctenopharyngodon idellus*) ovary cells when stimulated with biotin-tagged poly dG/dC [37].

The gene *psmb13a* (proteasome 20S subunit beta 8A), one of the subunits that make up the proteasome essential for proper protein turnover, quality control, and cellular function, was upregulated in shad exposed to CAP in response to oxidative stress at 96 h. Similarly, it implies increasing protein misfolding, mislocalization, and accumulation of toxic protein aggregates, contributing to various cellular dysfunctions. The glycerophospholipid metabolism pathway is a biochemical process involved in the synthesis, modification, and degradation of glycerophospholipids, which are a major class of phospholipids found in cellular membranes. Glycerophospholipids play essential roles in maintaining membrane structure, cell signaling, and lipid homeostasis [38]. Phospholipase A1A (*pla1a*, positive with MDA contents, histological changes) is essential for maintaining membrane integrity and lipid homeostasis. An enzyme that belongs to the phospholipase A1 family was upregulated, indicating a suppression in elevating stress such as oxidative stress and endoplasmic reticulum stress in shad caused by CAP. The gene *shmt2* (serine hydroxymethyltransferase 2) is an important enzyme that plays a crucial role in maintaining cellular folate homeostasis and regulating the availability of one-carbon units [39]. The downregulation of *shmt2* (positive with MDA contents, histological changes) in fish exposed to the pesticide CAP in this study can lead to a reduction in the biosynthesis of certain essential amino acids, such as glycine and serine, due to disruptions in one-carbon metabolism, nucleotide biosynthesis, and epigenetic regulation.

CAP caused the upregulation of *ppa2* (inorganic Pyrophosphatase 2). ppa2 catalyzes the hydrolysis of inorganic pyrophosphate (PPi), an enzyme essential for maintaining an appropriate balance between pyrophosphate and phosphate levels, which is crucial for cellular energy metabolism and homeostasis. The upregulation of *ppa2* at 96 h indicated efficient energy production, while prolonged exposure caused a downregulation at 192 h. Accumulation of PPi can also lead to increased oxidative stress and the generation of reactive oxygen species (ROS). The gene *pkc*–*β* (mTOR signaling pathway), an isoform of the protein kinase C (PKC) family of serine/threonine kinases, was downregulated, leading to impaired mTOR activation, compromised metabolic regulation, reduced cell growth and proliferation, altered stress responses, and increased susceptibility to apoptosis. The gene *diablo*, a pro-apoptotic protein that plays a crucial role in the regulation of programmed cell death, was involved in the apoptosis pathway. The gene *bcl2l1*, an anti-apoptotic protein that helps prevent programmed cell death (apoptosis) by inhibiting the activation of pro-apoptotic proteins, was downregulated in response to CAP exposure, making the cells more susceptible to undergoing apoptosis. A similar effect of CAP on apoptosis was found in crucian carp exposed to sublethal concentrations [8]. Genetic deletion of *hspa8* leads to selective tissue malformations in zebrafish’s embryonic development [40]. The NOD-like receptor signaling pathway is a critical component of the innate immune system, associated with the regulation of oxidative stress and the activation of antioxidant defense mechanisms, potentially influencing the response to pesticide-induced oxidative damage. The gene *stat1b* plays a crucial role in the body’s immune response, and it was upregulated under this pathway in response to CAP exposure.

The transcriptome can help readers identify DEGs that are affected in many signaling pathways, and the Environmental Protection Agency’s Sequence Alignment to Predict Across Species Susceptibility (SeqAPASS) tool [18] can also help us with the extrapolation of toxicity knowledge across over 100 fish species. The threshold [17,18,19,20] may be important for the number of DEGs, which may be different among different species [22] and different sizes, like juvenile and adult fish [21]. Although chemicals have a significant impact on juveniles, for adults, this significant impact may persist and be accompanied by other effects [23], which has been confirmed in white shrimp [20,21], zebrafish [23], fathead minnows [18], yellow perch [17], largemouth bass [24], Baikal Whitefish [19], rainbow trout [41]. The differences in metabolic activities and cell-line-specific toxicity via cell lines [42] will differ between juvenile and adult fish, which has been demonstrated in a fish model, zebrafish [43,44]. The precise mode of action needs to be elaborated via further gene knockout or inhibitor validation experiments [45,46,47].

### 3.3. Public Health for CAP Through Food Chains

CAP can be found in water and sediment samples in some South American countries with higher CAP production [4,12,25], showing the threat to aquatic organisms in different ecological niches [26]. The issues of CAP residues for animal health in wild and farmed shad will attract the attention of many researchers, because shad is delicious and economically valuable in the modes of the recirculating aquaculture system and photovoltaic farming. Pest control after such pesticide application can lead to potential contaminations of soil or surface water due to the flow of water in rice fields, spray drift, and soil adsorption and could be a threat to human health [48]. Researchers reported the CAP determination method in fruits and vegetables (the limits of detection and quantification were 0.8 and 1.6 mg·kg^−1^) via liquid chromatography–electrospray tandem mass spectrometry [49] but 0.005 and 0.0152 μg·mL^−1^ in the water samples [50], and 6.49 ng with recovery rates of 83.04% to 98.50% for grape samples supplemented with more than 5 mg·kg^−1^ CAP [51]. Reports on the maximum permissible limits of CAP for human and/or animal consumption will provide basic data for dietary health evaluation, but, now, there are fewer reports found for CAP in fish tissues or their surrounding ponds, and there are also fewer reports on CAP’s toxicological effects in the early stages of fish hatchery.

### 3.4. The Limitations of This Research

The flaw of this study may not even not refer to this issue, or even the use of the whole body because of scale, which may be difficult for comparisons with adult shad livers to present the clear mode of action, but some speculated pathway, like the antioxidant defense system, has been demonstrated to be affected in juvenile and adult American shad after CAP exposure, but the AMPK signaling pathway, at least AMPK activities in this study, relative to adipogenesis and endoplasmic reticulum stress, has been shown to be significantly affected in 3T3-L1 cells after CAP exposure [52] but showed no significant change. The associated genes involved in this pathway and its biological justification need to be further studied via gene knockout, activator or inhibitor addition experiments, which may sometimes overstate the interpretation of transcriptomic data. Also, the ecological relevance and, fortunately, the concentration of CAP exposure were close to the contraction in the water sample in the rice–crab system in China. Though elevated oxidative stress was found both in juvenile and adult shad, its response was different. Another one is the time exposure duration; this study does not clearly show what happened between around 8 h and 192 h for juvenile shad and 2 h and 96 h for adult shad, and these two sets of parameters used for juvenile and adult shad cannot be compared simultaneously.

## 4. Materials and Methods

### 4.1. Experimental Design in Juvenile and Adult American Shad and Sampling

**Figure 9:**
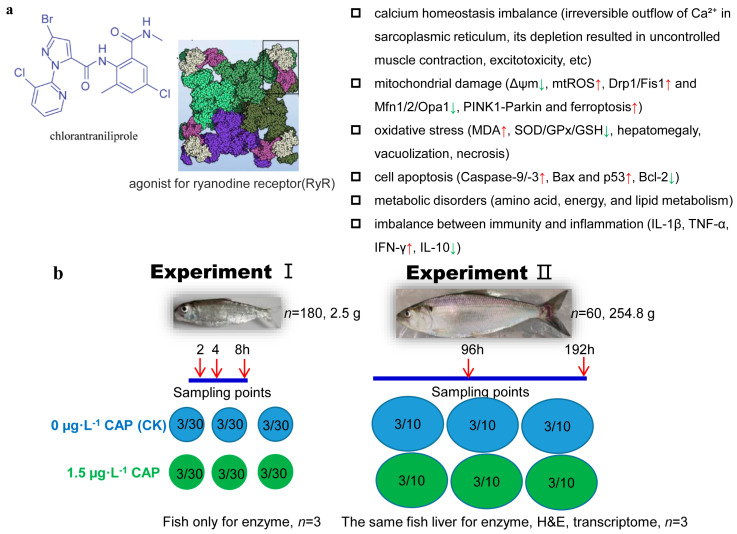
The toxicological mechanism of CAP (**a**) on fish and the experimental design (**b**). CAP is the agonist for RyR, which can result in six aspects of impact. “↓” and “↑” stand for down- and upregulation.

Juvenile (2.5 ± 0.2 g, 4.5 ± 0.4 cm, *n* = 180, Figure 9) and adult (254.8 ± 20.2 g, 22.5 ± 0.4 cm, *n* = 60) American shad, *A. sapidissima* (Wilson, 1981), samples were collected from two different indoor aquaculture ponds (juvenile and adult fish water condition, pH 8.5, temperature 28 °C, with dissolved oxygen 6.5 and 8.5 mg·L^−1^, respectively) at the Yangzhong Base of Freshwater Fisheries Research Center in Zhenjiang City (119°82′ E, 32°30′ N), Jiangsu Province. They were maintained and then transferred to independent tanks for CAP exposure treatments. CAP (C_18_H_14_BrCl_2_N_5_O_2_, CAS 500008-45-7, Invitrogen, Beijing, China, 1.2 mL, 100 μg·L^−1^, with 99% purity) solution, dissolved in DMSO (0.1%, Sigma-Aldrich, Beijing, China), was added to the water at a concentration of 1.5 μg·L^−1^ (the sensitive concentration ranged from 1.35 µg·L^−1^ to 34.43 mg·L^−1^ [4], also close to the safe exposure concentration, 1/10 96 h LC_50_ of 14 μg·L^−1^). Exposure concentrations were selected based on environmentally relevant values and exposure experiments reported in previous studies [4,12,17,18,25,26]. Throughout the experiment, fish were maintained independently in aerated water with controlled temperature and quality, using a recirculating culture system supplied by Guangzhou Degang Aquatic Equipment Technology Co., Ltd. (Guangzhou, China). Fish were fed a commercial diet without CAP purchased from Zhejiang Minghui Feed Co., Ltd. twice daily (Jiaxing, China, crude protein 4%, crude fat 6%). Water conditions met the Chinese fishery standards (GB11607-1989 of China) [53] and monitored every day, including a pH range of 6.9–7.8, dissolved oxygen levels of 5–8 mg·L^−1^, and temperature maintained at 28 ± 1 °C for two dependent experiments, and morphometric characteristics of the total length, total weight showed no significant difference in treatments and controls in both experimental sets. All procedures complied with institutional animal ethics guidelines (LAECFFRC-2023-07-14).

Experiment I randomly selected juvenile fish (*n* = 180, *n* = 30 for each tank and *n =* 90 for each treatment group in triplicate), introduced into six experimental fish tanks (Figure 9b, *n =* 3, 100 L, 1.5 μg·L^−1^ CAP, corresponding to a 96 h LC_50_ of 14 μg·L^−1^ for juvenile American shad, with a safe concentration of approximately 1.5 μg·L^−1^) and controls (*n =* 3, designated as CK) in triplicate. After the acute exposures for 2, 4, and 8 h, 3 whole fish were mixed (2~3 individuals to meet the total amount for enzymatic activity determination). Samples were randomly collected from each tank and immediately preserved in a freezer at −80 °C. Due to the limited time for indoor cultivation of shad, our previous study used 70 and 140 mg·L^−1^ enrofloxacin exposure for 12~48 h [14]. The time duration for juvenile shad selected followed a previous study of juvenile *Clarias gariepinus* (10.9 g, 175 mg·L^−1^) for 15 d [6], 0.3~30 mg·L^−1^ CAP exposure in juvenile crucian carp (20~25 g) for 14 d [8], 0.2 μg·L^−1^ CAP exposure in juvenile yellow perch (0.92 g) for 28 d [17], 10~250 μg·L^−1^ CAP exposure in 24 h post-hatch juvenile fathead minnow for 96 h [18]. Apart from pH, temperature, and dissolved oxygen, which were controlled via hydrochloric acid–sodium bicarbonate system, heating rod, and aerator, respectively, the main water quality, TN, TP, COD_Mn_ were determined at each sampling point and ranged around 2.3~3.2, 0.4~0.7, 16.2~24.3 mg·L^−1^, respectively.

To investigate the harmful effects for CAP on different stages of shad in Experiment II, randomly selected adult fish (*n* = 60, *n =* 10 for each tank and *n =* 30 for each treatment groups) were exposed to 1.5 μg·L^−1^ CAP for 96 and 192 h in six independent tanks (1500 L), and samples were collected for histopathological (*n =* 3 per treatment) and transcriptome (*n =* 3 mixed per treatment to meet 100 ng·μL^−1^) analysis. Our previous study used 70 and 140 mg·L^−1^ enrofloxacin exposure for 12~48 h [14], and 0.7 mg·L^−1^ copper sulphate exposure for 72~144 h in shad because of the challenge of lab culture [15]. The time duration for adult shad selected followed the half-life of 1000 days [9], and the previous study of 0.1 and 1 mg·L^−1^ CAP on four-mouth-old zebrafish for 21 d [4], 0.15 and 1.5 mg·L^−1^ (1/10 96 hLC_50_) CAP on adult female *Cnesterodon decemmaculatus* for 96 h [12]. For both the juvenile and adult fish, CAP concentrations used in this study mainly followed the detected CAP concentrations in USA (10.2 µg·L^−1^) [12], China (1.35 µg·L^−1^), and 0.2 µg·L^−1^ CAP used in the previous study of yellow perch [17], even though the used CAP concentrations were 1.5 µg·L^−1^. Liver samples (*n* = 3 mixed for treatments obtaining from the total 9 fish from the separate 3 triplicate tanks) from the same group were randomly collected for enzymatic activity determination, MDA content measurement, transcriptomic analysis (sampling liver from the same fish, from groups labeled CB96 and CB192 for controls, NB96 and NB192 for CAP exposure groups), histopathological assessment (sampling liver from the same fish). Before all procedures, fish were euthanized with tricaine methanesulfonate (MS-222, 50 mg·L^−1^, Sigma-Aldrich, St. Louis, MO, USA) to minimize stress and ensure humane handling.

### 4.2. Enzymatic Activities, MDA Contents for Juvenile American Shad

In Experiments I (2.5 g) and II (254.8 g), the difficulty of separating detoxifying organ liver tissue varied depending on the different specifications of shad. In the case of juvenile fish, this study only used the whole body for analysis and cannot compare and analyze with the liver tissue results in adults. For biochemical analyses, the whole body (*n =* 3 mixed per treatment) per group at every sampling point was washed with ice-cold physiological salt water (0.86% NaCl) thoroughly; then, we dried the surface with absorbent paper and weighed. For biochemical and enzymatic assays at 2, 4, and 8 h (exposure time was according to [14]), 0.5 g of the whole body was homogenized in ice with cold 0.86% physiological salt water (1:9, *w*/*v*) and then centrifuged at 2500 r/min at 4 °C for 10 min. The corresponding supernatants were used for the detection of enzymatic biomarkers. All biochemical analyses, including normalization procedures for both samples and reference standard, were conducted using commercial kits from Nanjing Jiancheng Bioengineering Institute (Nanjing, China), following the manufacturer’s instructions and previous experimental references [4,14,15]. The enzymatic contents that were analyzed were according to our previous studies [14,15], including acid phosphatase (ACP, A060-2-2, wavelength = 530 nm), alkaline phosphatase (AKP, A059-2-2, wavelength = 530 nm), polyphenol oxidase enzyme (PPO, A136-1-1, wavelength = 420 nm), 7-ethoxy-3-isophenoxazolone-deethylase (EROD, MM-91756O1, wavelength = 450 nm), aryl hydrocarbon receptase (AHR, MM-925584O1, wavelength = 450 nm) [13,14,15], and ryanodine receptase (RYR, K6-14356, wavelength = 450 nm) [13,14,15], adenosine 5′-monophosphate (AMP)-activated protein kinase (AMPK, H355-1-2, wavelength = 450 nm, µg·mgprot^−1^), tumor necrosis factor alpha (TNFα, H052-2-2, wavelength = 450 nm, ng·mgprot^−1^) [13]. The trace malondialdehyde (MDA, A003-2-2, wavelength = 532 nm) contents were simultaneously determined [4]. Biochemical parameters were quantified using a spectrophotometer (Jasco-V530, Beijing, China), with absorbance measured within a wavelength range of 420 to 532 nm, depending on the specific reagent and target compound.

### 4.3. Histological Slice for Adult American Shad

The liver samples (*n =* 3 per treatment) for 96 and 192 h (exposure time was according to [14,15,26]) were embedded in paraffin (Leica RM2235, Leica Microsystems, Vizna, Germany), sectioned, and stained with hematoxylin and eosin (H&E) using standard techniques and subjected to microscopic examination using light microscopy. The samples were examined using a compound microscope (400×, Olympus CHC binocular, Tokyo, Japan), and digital images were taken of each of the liver samples. The qualitative changes were performed first, and then quantification of the histological changes when compared with the controls, the coverage areas with certain characteristic changes, like vacuolation, hydropic degeneration, were statistically analyzed using quadrat methods from NanoZoomer^®^S360 (Hamamatsu Photonics, Hamamatsu City, Japan), with 10 slices for each shad individual in triplicate.

### 4.4. Enzymatic Activities, MDA Contents and Cytokines for Adult American Shad

For biochemical analyses (*n =* 3 mixed per treatment) in adult American shad liver after the normalization procedures above, the liver per group at every sampling point was determined, as well as for the indices, the enzymes (ACP, AKP, PPO, EROD, AHR, RYR, AMPK, TNFα) and MDA contents.

### 4.5. Transcriptome Analysis in Adult American Shad

Total RNA (*n =* 3 mixed per treatment) for 96 and 192 h was extracted (TRIzol^®^ Reagent, Invitrogen, Beijing, China), and genomic DNA was removed using DNase I (TaKara, Beijing, China). RNA quantification and qualification were performed using the Qubit R RNAAssay kit on Qubit R 2.0 Fluorometer (Life Technologies, Carlsbad, CA, USA) and Agilent Bioanalyzer 2100 system (Agilent Technologies, Santa Clara, CA, USA). RNA integrity was evaluated (RNA Nano6000 detection kit, Agilent Bioanalyzer 2100 system, Santa Clara, CA, USA), the RNA concentration was determined (American Life Technology Company, Carlsbad, CA, USA), and samples with RNA integrity number values larger than 7 were selected.

A total of 1 μg RNA was used for each sample as input material for RNA sample preparation. The mRNA was purified from total RNA using poly–T oligomagnetic beads, the mRNA was broken into short fragments by adding fragmentation buffer, the first strand cDNA was synthesized with six base random primers using mRNA as a template, and then the second-strand cDNA was synthesized with DNA polymerase I and RNA enzyme H. The remaining overhangs were converted to blunt ends by exonuclease/polymerase. After adenosylation at the 3′ end of the DNA fragment, hybridization was carried out in combination with the nebnext adapter with a hairpin ring structure. In order to preferentially select cDNA fragments with a length of 200~250 bp, they were purified using the ampurexp system (Beverly, Los Angeles, CA, USA). Then, we used 3 μL user enzyme (Invitrogen, Carlsbad, CA, USA), 37 °C adapter connection cDNA 15 min, 95 °C 5 min. Fusong high-fidelity DNA polymerase, universal PCR primers and index primers were used for PCR research.

To ensure the quality of transcriptome analysis, the original sequencing data were filtered (raw reads), the reads with connectors were removed, and the reads with a proportion of N (N indicates that the base information cannot be determined) greater than 0.1% and reads with low quality (the base number with quality value Qphred ≤ 20 accounted for more than 50% of the whole reads) produced high-quality sequencing data (clean reads). Trinity v2.15.1 (Broad Institute, Boston, MA, USA), BUSCO version 5.7.0 (Benchmarking Universal Single-Copy Orthologs, Cambridge, MA, USA), TransDecoder v 5.7.1 (GitHub Support from Johns Hopkins University, Baltimore, MD, USA) software were used to splice clean reads, perform quantitative assessment, obtain non-redundant transcript sets, respectively, and the method for de novo assembly, reference genome selection (GCA_018492685.1) was according to a previous study [19,20]. Functional annotations were performed according to previous studies [26,29].

The fragments per kilobase of transcript per million fragments mapped (FPKM) method was used to calculate the levels of gene expression, and DESeq2 v1.30.1 was used to analyze the differences in gene expression across treatment groups. Differentially expressed genes (DEGs) were found and selected using the following criteria: |log_2_ (fold change)| > 1 and adjusted *p*-value (*q*-value) of less than 0.05. With adjusted *q* < 0.05 as the cutoff, a Gene Ontology (GO) and Kyoto Encyclopedia of Genes and Genomes (KEGG) enrichment analysis of these DEGs (threshold *p*-value 0.05 and fold change ±2) was performed using the Cluster Profiler tools in R software version 4.1.0. To identify the affected genes under CAP exposures, we screened significant DEGs (threshold *p*-value 0.05 and fold change ±2) in the KEGG pathways associated with ribosome, proteasome, oxidative phosphorylation, ferroptosis, spliceosome, biosynthesis of amino acids, glycerophospholipid metabolism, mTOR signaling pathway, peroxisome, protein processing in endoplasmic reticulum, NOD-like receptor signaling pathway, apoptosis, necroptosis. Variations in significant increases or decreases were constructed through a series of comparisons (e.g., CB96 vs. NB96, CB192 vs. NB192, and NB96 vs. NB192).

### 4.6. Validation of Data via qPCR in Adult American Shad

The specificity test for RNA-Seq analysis was performed using the qPCR method for 96 and 192 h [14,15,17,18,26]. qPCR (*n* = 3 mixed per treatment) was used to validate DEGs involved in the KEGG pathways related to ribosome, protein processing in the endoplasmic reticulum, glycerophospholipid metabolism, biosynthesis of amino acids and mTOR signaling pathway. This study selected *β-actin* as the stable reference gene (reported in shad in refs. [14,15]) based on the stability of the 4 reference genes (other 3 were *eef1a*, *gapdh*, and *tuba1*) assessed following our previous study performed in crucian carp within the standard MIQE guidelines [29], computed changes in mRNA levels via 2^−ΔΔCT^ method (*n* = 3).

Complementary DNAs (cDNAs) were synthesized from 3 μg total RNAs with M-MLV reverse transcriptase (Sigma-Aldrich, Beijing, China) and oligo(dT)_18_ primer in 20 μL final volume. Primer sequences used for qPCR analysis using CFX96 thermocycler (Bio-Rad, Hercules, CA, USA) and SYBR Premix ExTaq II kit (TaKaRa, Beijing, China) are listed in Table 1. The qPCR reactions were carried out in a final volume of 25 μL, using 1 × SYBR *Premix Ex Taq™* (Takara, Dalian, China), 0.4 μM of each primer, and 2.5 μL RT reaction solution. Cycling parameters were as follows: initial denaturation at 95 °C for 30 s, followed by 40 cycles of denaturation at 95 °C for 5 s, and annealing/extension at 60 °C for 30 s. Each sample was run in triplicate. A melt curve analysis was performed at the end of each PCR thermal profile to verify the specificity of each amplicon. The efficiency (E) of each PCR was determined by the slope generated using a 10-fold diluted cDNA series with five dilution points in triplicate. The equation was *E* = 10^(−1/slope)^.

### 4.7. Statistical Analyses

The data were presented as the mean ± SD. To determine statistical differences between the CAP treatment groups and the control group, NDP.view 2.9.22 RUO from NanoZoomer^®^S360 (for H&E) and SPSS 26.0 (IBM, Armonk, NY, USA) was used. Prior to statistical testing, data that did not conform to normal distribution or homogeneity of variance were log_2_-transformed to meet the assumptions of parametric analysis. *T*-test was used to assess differences among treatment groups at the same sampling points. One-way ANOVA is used to compare differences at different times in controls and shown with different lowercase letters. Additionally, graphical illustrations were generated using GraphPad Prism version 10.0 (GraphPad Software, San Diego, CA, USA). A *p*-value less than 0.05 was considered statistically significant. A principal component analysis (PCA) was performed to observe potential clusters of the enzyme biomarkers evaluated by the effect of CAP on the whole body and liver of shad between treatments, and redundancy analysis (RDA) was conducted to observe whether the enzyme biomarkers can be affected when using CAP concentrations as variable environmental factors.

## 5. Conclusions

The inhibition of metabolic enzymes (AKP, PPO) and inflammatory cytokine (TNFα) at the protein level at 8 h for juvenile and adult shad at 96 and 192 h showed an increase in the oxidative stress biomarker, MDA (8 h for juvenile, 96 h and 192 h for adult) contents, metabolic enzymes, ACP (2~4 h), AHR (2 h), and EROD (8 h) for detoxification at the protein level for juvenile shad when exposed to CAP, which demonstrated CAP caused oxidative stress, metabolic and detoxification disorders, and inflammation in juvenile and adult American shad. For adult shad, there were CAP-induced changes in the liver, which became more pronounced over time. This change confirms the results of oxidative stress, as detected through changes in enzyme activities. Concurrently, there were changes in the expression of genes related to translation, folding, sorting and degradation, energy metabolism, cell growth and death, and the immune system. Particularly, the ribosome, protein processing in the endoplasmic reticulum, apoptosis, and necroptosis were the most significantly affected pathways. These findings indicated that this sublethal concentration of CAP may exert significant oxidative stress, metabolic and detoxification disorders, and inflammation in juvenile and adult American shad. The differences in the response of different specifications of shad to CAP also remind us to pay attention to the continuous monitoring of Bisamide antibiotic concentrations throughout the development and breeding process of shad and to find mitigation strategies from Chinese herbs and active substances. In addition, the limitations of the experimental design and statistical analysis in this study caused confusion for readers. Although it is not possible to standardize the comparison between whole body and live samples for both juvenile and adult shad, and there is no joint comparison of concentration and exposure time duration, many conclusions are speculative, and it is difficult to clarify the precise toxic effects of CAP on shad. Such issues can be addressed in future research.

## Figures and Tables

**Table 1:** The qPCR primers used in the present study.

Pathway	Gene	Accession Nuber	Primer Efficiency	Tm (°C)	Primers	Product (bp)
reference gene	*β-actin*	XM_042110509.1	98%, *R*^2^ = 0.9956	60	F: GTGGATCAGCAAGCAGGAGT R: ATCCTGAGTCAAGCGCCAAA	165
ribosome	*rpl34*	XM_042070778.1	99%, *R*^2^ = 0.9875	59	F: CCTCTGTCGTGGGGTTTTCA R: GGCCTTGCCTGTTTTCTTGG	202
protein processing in the endoplasmic reticulum	*capn2*	XM_042105064.1	97%, *R*^2^ = 0.9913	60	F: CCCCTGCCCATTCAAAGGAT R: TTCGAAGAGAGTGCCGCTTT	266
mTOR signaling pathway	*prkcb*	XM_042104217.1	95%, *R*^2^ = 0.9928	60	F: GACAAAGGACCAGCGTCAGA R: GACCGTGAGTGTGTCGTTCT	258
glycerophospholipid metabolism	*pla1a*	XM_042068648.1	94%, *R*^2^ = 0.9901	60	F: CTGTGCCAACCTGTTTACGC R: GAGGCGCTGTAGATCCAGTC	201
biosynthesis of amino acids	*shmt2*	XM_042088874.1	96%, *R*^2^ = 0.9947	60	F: ACATGGCTCACATCAGTGGG R: TCTCACGGCCCTTCTTTTCC	165

## Data Availability

The original contributions presented in this study are included in the article/Supplementary Materials. Further inquiries can be directed to the corresponding authors.

## Supplementary Materials

The following supporting information can be downloaded at: https://www.mdpi.com/article/10.3390/ijms27125383/s1.
